# Purified Saponins in *Momordica charantia* Treated with High Hydrostatic Pressure and Ionic Liquid-Based Aqueous Biphasic Systems

**DOI:** 10.3390/foods11131930

**Published:** 2022-06-29

**Authors:** Jing Ma, Hongkai Yang, Yajing Chen, Xiaoping Feng, Chunyu Wu, Fangyu Long

**Affiliations:** College of Food Science and Engineering, Northwest A&F University, Xianyang 712100, China; majing1119436969@nwafu.edu.cn (J.M.); kai.hong@foxmail.com (H.Y.); jeanchen@nwafu.edu.cn (Y.C.); xiaoping-feng@nwafu.edu.cn (X.F.); wcyup304424@163.com (C.W.)

**Keywords:** high hydrostatic pressure, ionic liquids, aqueous biphasic systems, *Momordica charantia* L., saponins

## Abstract

*Momordica charantia* L. (Cucurbitaceae) is rich in saponins, which have multiple biological effects. In this study, the total saponins of *M. charantia* were extracted by high hydrostatic pressure (HHP) technology. The optimal extraction process was determined (ethanol concentration 68%, pressure-holding time 8 min, ratio of material to solvent 1:35 and pressure 510 MPa), and the extraction amount of saponins reached 127.890 mg/g. On this basis, an ionic liquid-based aqueous biphasic system was constructed to purify the total saponins. Under the optimized conditions, the purity of *M. charantia* saponins was 76.06%. Liquid chromatography–mass spectrometry (LC/MS) was used to characterize the saponins in the purified extract of *M. charantia*. It was found that there were four kinds of saponins in the extract of *M. charantia*: kuguaglycoside A, momordicoside L, kuguacin B and kuguacin J, providing a basis for the study of the biological activity of saponins.

## 1. Introduction

*Momordica charantia* L. (Cucurbitaceae), commonly known as bitter gourd or bitter melon [1], is commonly cultivated in India, China, and Central and South American countries [2]. *M. charantia* contains saponins, proteins, polysaccharides, polyphenols, steroids, and other bioactive components [3]. Among them, saponins have antiinflammatory, antidiabetic, immune enhancement, anticancer and other biological activities [4,5]. It has been proposed that these therapeutic effects are associated with extracts prepared from *M. charantia* [6]. Extracts of *M. charantia* have been reported to depress the level of plasma glucose [7].

At present, the extraction of total saponins mainly adopts solvent extraction [8], ultrasonic [9], microwave-assisted extraction [10], reflux extraction [11] and other techniques [12]. Traditional extraction methods such as heat reflux extraction and maceration extraction may be time-consuming and require relatively large quantities of solvent [11]. The high hydrostatic pressure (HHP) technique uses a controllable temperature in the process of extraction, which is beneficial to the study of biological activity and reduces the loss of volatile components [13] and the effect of temperature on biological activity. Recent applications of HHP technology have been broadly used in the extraction of saponins [14], flavonoids and polysaccharides [15], and polyphenols [16]. It has been suggested that high pressure can cause cell deformation, cell membrane damage and even cell rupture, thereby allowing the contents to ooze out [17]. Therefore, the optimal process conditions for the extraction of saponins were investigated, which could provide the basis for the study of the biological activity of saponins in *M. charantia*.

The extracted solutions generally contain many other chemical constituents as well as saponins; in order to ensure the pharmacological activities of the extracted solutions, a feasible method to prepare high-purity total saponins is needed [18]. At present, the most commonly used methods for the purification of saponins include macroporous resin adsorption, aqueous biphasic systems, liquid–liquid extraction and membrane separation. Ionic liquid (IL) is a liquid salt with low volatility and high stability [19]. What is more, IL can be designed by combining multiple cations and anions to allow for the efficient extraction of different target compounds. Compared to traditional purification methods, IL technology is simple to operate, and is fast and efficient. Ionic liquids have been used to extract aromatics from aliphatics [20] and rare-earth elements [21]. The structure of IL is closely related to its physical and chemical properties, which determine its performance in purification. Due to its strong ability to destroy cell walls and dissolve compounds of imidazole-type IL, it is often used to extract small molecule natural products from herbal medicines [22]. In addition, imidazole-type IL usually has a high phase separation capacity owing to its low density charge. Aqueous biphasic systems (ABS) have been used in previous studies to concentrate or purify extracts [12]. Huang et al. [23] used alcohol/K_2_HPO_4_ ABS to concentrate glycyrrhizin from licorice extract. Nevertheless, the purity of the resulting triterpenoid saponins was low due to the interference of phenolic compounds. Ionic liquid-based aqueous biphasic systems (IL-based ABS) were first developed by Gutowski, Broker and Willauer [24], and have been applied as an effective method for the preconcentration or purification of a wide range of compounds [25]. Ji et al. [12] replaced the inorganic salt fraction in IL-based ABS with sodium hydroxide to produce deprotonated (ionized) forms in an acid-base neutralization reaction, which can purify triterpenoid saponins. IL-based ABS has received more attention than conventional polymer ABS due to its adjustable extraction capability, easily achievable phase separation and mild operating conditions. Nevertheless, no purification of *M. charantia* saponins by using IL-based ABS has been reported.

The technology for preparing *M. charantia* powder is relatively simple and well established. Popovich et al. [26] removed the seeds and cut *M. charantia* into small pieces, then lyophilized these to prepare *M. charantia* powder. Tan et al. [10] used a similar method to prepare *M. charantia* whole fruit powder. Thus, we utilized purchased *M. charantia* powder to investigate the effect of the HHP auxiliary extraction treatment on the production of total saponins from *M. charantia.* In addition, we report here the procedure of IL-based ABS, which was used for the purification of the extracted *M. charantia* and to optimize the purification conditions. Finally, liquid chromatograph–mass spectrometry (LC/MS) was performed to identify the composition of the extracted solutions after being purified.

## 2. Materials and Methods

### 2.1. Chemicals

The standard of dioscin and 1-butly-3-methylimidazolium tetrafluoroborate ([C_4_MIM]BF_4_, ≥97.0%, CAS: 174501-65-6) were purchased from Solarbio (Beijing, China). Vanillin (≥99.0%, CAS: 121-33-5) was obtained from Tianjin Kemiou Chemical Reagent Co., Ltd. (Tianjin, China). Chromatographic grade methanoic acid (≥98.0%, CAS: 64-18-6) and acetonitrile (≥99.9%, CAS: 75-05-8) were obtained from Sigma-Aldrich-Fluka (Shanghai, China) and used for high performance liquid chromatography (HPLC) analysis. Other kinds of solvent used in the experiment, such as ethanol, were analytical grade and purchased from Sichuan Xilong Chemical Works (Chengdu, China). *M. charantia* powder (*M. charantia* pericarp and inner tissue were dehydrated, then vacuum dried) was purchased from Hunan Changsha Bojian Biological Technology Co., Ltd. (Changsha, China).

### 2.2. Extraction of Total Saponins

#### 2.2.1. Single Factor Experiments for Extraction

The *M. charantia* power was passed by 60 mesh sieve used for analysis. High hydrostatic pressure equipment (HHP 600 MPa/10 L, Baotou Kefa High Pressure Technology Co., Ltd., Baotou, China) with a pressure vessel volume of 10 L was used for high pressure treatment. The unit has an output pressure range of 0 to 600 MPa. The pressure come-up time using this system was approximately 90 s and the pressure release was an average of 20 s. Processed samples were transferred into a sterile polyethylene bag and heat-sealed under a vacuum. The bags were placed into the pressure vessel and treated in different conditions. In this experiment, the output of total saponins from *M. charantia* was used as an evaluation index, and a 4-factor, 7-level scheme was used to screen the appropriate range. The four factors with seven levels were as follows [13,15], ethanol concentration (55%, 60%, 65%, 70%, 75%, 80%, 85%), pressure-holding time (2, 4, 6, 8, 10, 12, 14 min), material to solvent ratio (1:15, 1:20, 1:25, 1:30, 1:35, 1:40, 1:45 g/mL) and pressure (300, 350, 400, 450, 500, 550, 600 MPa).

#### 2.2.2. Box–Behnken Design for Extraction Process

According to the results Table S1, three levels for pressure, pressure-holding time, ethanol concentration and material to solvent ratio were selected for Box–Behnken design (BBD), and the experimental schemes of BBD were described with twenty-four factorial points and five replicates of central points.

### 2.3. Purification of Total Saponins

#### 2.3.1. Single Factor Experiments for Purification

The total saponins from *M. charantia* were purified using methods adapted from the literature with minor modifications [12]. *M. charantia* powder (0.5 g) was added into the IL aqueous solutions ([C_4_MIM]BF_4_), sodium hydroxide was added and the mixture was vortexed until the two phases were separated, and then, centrifuged for 5 min at 1800× *g*. In order to obtain the optimal [C_4_MIM]BF_4_ concentration, the concentration of sodium hydroxide and the material to solvent ratio for purification of total saponins by IL-based ABS, a series of single factor experiments were conducted. The [C_4_MIM]BF_4_ concentration range from 0.5 to 2.5 M was performed to investigate the influence of [C_4_MIM]BF_4_ concentration on the extraction efficiency of total saponins, with the same sodium hydroxide concentration and material to solvent ratio. Likewise, the sodium hydroxide concentration (6–14%) and material to solvent ratio (1:6–1:14 g/mL) were also explored, respectively. 

#### 2.3.2. Box–Behnken Design Optimization for Purification Process

A response surface methodology with the Box–Behnken system was employed to design an experiment consisting of 17 runs with 12 factorial points and 5 central points. The independent parameters were [C_4_MIM]BF_4_ concentration (A), material to solvent ratio (B) and sodium hydroxide concentration (C), and the chosen code variable levels for each were based on the preliminary experiments (Table S2). To express the purity as a function of the independent variables, a 2nd-order polynomial equation was generated as follows

Y_i_ = a_0_ + a_1_A + a_2_B + a_3_C + a_11_A_2_ + a_22_B_2_ + a_33_C_2_ + a_12_AB + a_13_AC + a_23_BC (1)

where Y is the dependent response (purity); A, B and C are the levels of the independent variables [C_4_MIM]BF_4_ concentration, material to solvent ratio, and sodium hydroxide concentration, respectively, and a_0_, ai, aii and aij are the regression coefficients of the variables for the offset, linear, quadratic and interaction terms, respectively.

### 2.4. Determination of the Total Saponins Content

Total saponin content from *M. charantia* extracts was carried out by enzyme-labeled instrument (PerkinElmer, Waltham, MA, USA) according to the method described by Perez et al. [8]. Ethanol was used as solvent to prepare different concentrations of standard dioscin (from 0 to 300 μg/mL), then a standard curve was drawn. The reaction consisted of the addition of 250 µL of standard dilution to 8% vanillin–acetic acid solution (250 µL). After vortexing, 72% sulfuric acid aqueous solution (2.5 mL) was added to the reaction mixture followed by incubation at 60 °C in a water bath for 10 min. The sample was cooled to room temperature and absorbance values were recorded at 544 nm. The total saponins’ content of extracts was expressed as diosgenin equivalent (DE) mg/g of freeze-dried extracts.

The total saponins’ extract of 1.0 g *M. charantia* power was concentrated at 40 °C (90 r/min) until there was no visible smell of ethanol and no bubbles. Concentrated extract was subsequently lyophilized prior to analysis, then redissolved with ethanol to 10 mL. Ethanol was used to replace the sample as control, and the content of total saponins was determined according to the above method. The estimation of total saponin content was calculated according to the standard graph. All the colorimetric experiments were performed in triplicate. 

### 2.5. Purification of Total Saponins from M. charantia

**Figure 1:**
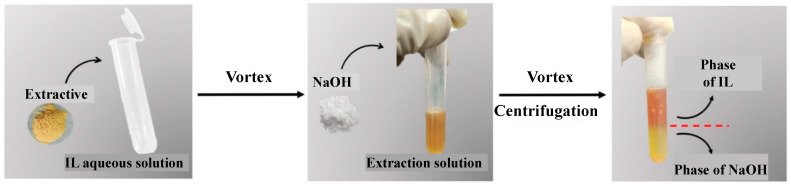
Flow chart for extraction and purification of total saponins from *Momordica charantia* L. by ionic liquid-based aqueous biphasic systems (IL-based ABS) based high hydrostatic pressure (HHP) extraction.

The purification of extracted solution from *M. charantia* was determined using method with minor modifications [12]. Figure 1 illustrates the process flow combining extraction and purification of total saponins from *M. charantia* by IL-based ABS-based high hydrostatic pressure extraction. A total of 0.5 g *M. charantia* powder was added to the aqueous solution of IL ([C_4_MIM]BF_4_) according to a certain ratio of material to solvent. After vortexing at room temperature, a certain concentration of sodium hydroxide was added, and continued to vortex until the two phases were separated. Subsequently, centrifuging for 5 min at 1800× *g*, the sodium hydroxide-rich lower phase was completely isolated from the IL-rich upper phase. 

### 2.6. Calculation of Extraction Efficiency and Purity of Total Saponins

The lower phase was lyophilized, then redissolved with ethanol. The content of total saponins from *M. charantia* was determined by the same method as Section 2.4, and the extraction efficiency (E, mg/g) and purity of saponins (P, %) were calculated as follows:

(2) $$E=\frac{X}{m};\;P\left(\%\right)=\frac{X}{M}\times 100\%$$

where m (g) is the weight of powder of *M. charantia.* X (mg) and M (mg) are the content of total saponins in sodium hydroxide-rich lower phase and weight of freeze-dried total saponins powder, respectively.

### 2.7. LC/MS Conditions and Parameters

Under optimal extraction and purification conditions, sample solutions were prepared with 10.0 mg of total saponins, and diluted to 10 mL with chromatographic methanol, then filtered through 0.22 μm membrane for further experiments. 

LC/MS analysis was performed on Triple quadrupole Tandem Mass Spectrometer (Qtrap 5500, SCIEX, Framingham, MA, USA). The chromatographic and MS conditions were determined by method described by Perez et al. [8] with some modification. The main chemical components of saponins were separated on a C_18_ chromatographic column (ZORBAX SB-C18, 250 mm × 4.6 mm, 5 μm, Agilent Technologies Inc., Santa Clara County, CA, USA) at flow rate 1.0 mL/min and detection with the wavelength 208 nm. The mobile phase consisted of 0.1% formic acid in water (solvent A) and 0.1% acetonitrile solution of formic acid (solvent B). The gradient elution was as follows: 10–70% B from 0 to 15 min, 70–100% B from 15 to 20 min, 100% B from 20 to 42 min. The LC/MS operating conditions were as follows: positive ion modes, scan with a scanning range of 400–850 *m*/*z*, drying gas with 600 L/h and 400 °C, and a capillary voltage of 3000 V. Comparing the accurate masses obtained from mass spectrometry data with theoretical values obtained from the literature, the accuracy between measured and theory data was reported as Δppm. The Δppm is calculated as follows:

(3) $$\Delta \mathrm{ppm}=\left({\mathrm{mass}}_{\exp}-{\mathrm{mass}}_{\mathrm{calc}}\right)/{\mathrm{mass}}_{\exp}\times 10^{6}$$

where mass_exp_- is the experimental mass and the mass_calc_- is the mass calculated from molecular formulas obtained from the literature. If Δppm values are within ±50 ppm, the compound is considered a positive match.

### 2.8. Statistical Analysis

Statistical analysis was performed using SPSS 20.0, *p*-value < 0.05 was considered statistically significant and *p*-value < 0.01 was considered highly significant.

## 3. Results and Discussion

### 3.1. Single Factor Experiments of Extraction Conditions

#### 3.1.1. Pressure

**Figure 2:**
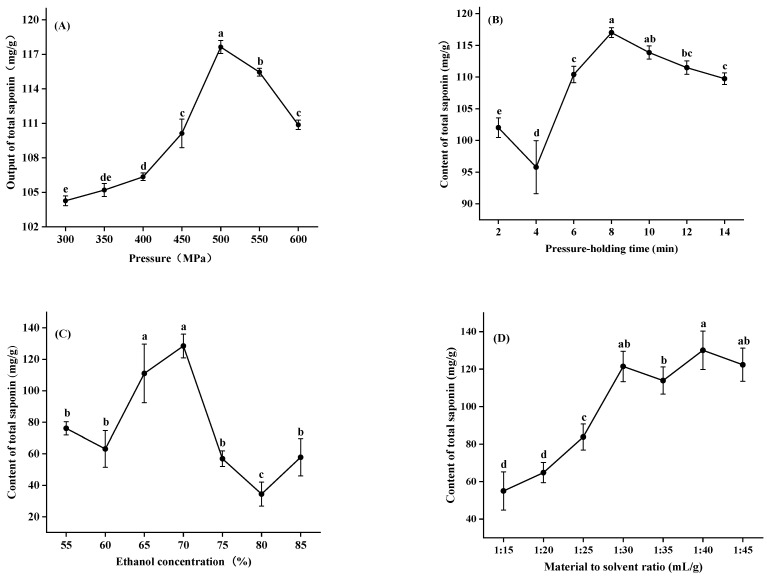
Effect of pressure (**A**), pressure-holding time (**B**), ethanol concentration (**C**) and material to solvent ratio (**D**) on the output of total saponins for HHP extraction. Values are means of triplicate ± standard deviation. Different letters represent significant differences (*p* < 0.05).

As can be seen from Figure 2A, the output of total saponins obtained by the HHP method shows a similar trend with the pressure when the extraction conditions are fixed to a holding time of 8 min, the solvent is 70% ethanol and the ratio of material to solvent is 1:30. Under constant conditions, a higher pressure helps to increase the extraction output. For instance, when the extract pressure increased from 300 MPa to 500 MPa, the output of total saponins distinctly increased and the maximum extraction output was achieved (117.629 mg/g). It is worth noting that the output of total saponin decreased rapidly when the extraction pressure was higher than 500 MPa (*p* < 0.5). This is because higher pressure treatment not only affects the structure of cells, but also affects the speed of the solvent entry to cells and the intracellular substance release [13]. The decrease in total saponin content may be due to the excessive pressure; the cell structure tends to be close, which prevents the saponins’ dissolution.

#### 3.1.2. Pressure-Holding Time

The influence of different pressure-holding times on the output of total saponins is presented in Figure 2B. Increasing the pressure-holding time from 2 to 8 min gradually, the output of total saponins increased significantly, and the maximum was achieved at 117.006 mg/g. When the extraction time was longer than 8 min, the output decreased gradually. The results showed that 8 min was enough to extract total saponins by the HHP method. Compared with enzymatic and ultrasonic extraction techniques, HHP extraction technology has a superior extraction time and extraction efficiency [27].

#### 3.1.3. Ethanol Concentration

Adding water into ethanol could improve the extraction efficiency of ginsenosides [13]. The effects of ethanol concentration in the extracted solvent on the output of total saponins from *M. charantia* are given in Figure 2C. When ethanol concentration increased from 55% to 70%, the content of total saponins gradually increased and achieved its maximum (128.502 mg/g). When the ethanol concentration was greater than 70%, the output of total saponins decreased quickly. This may be due to the fact that the protein coagulates in a high concentration of ethanol, and large diffusion resistance may limit the extraction of total saponins from *M. charantia* [8]. The optimal ethanol concentration for the total saponins’ extraction was 70%, which is in agreement with the results of Chen et al. [13].

#### 3.1.4. Material to Solvent Ratio

In general, for a fixed amount of raw material, the extraction output is promoted when the solvent volume is increased. The influence of the solvent to raw material ratio on the output of total saponins was evaluated (Figure 2D). With the increase in the ratio of material to solvent from 1:15 to 1:30 in the extraction system, the output significantly increased. This is because the increasing ratio of material to solvent could decrease the solution’s concentration difference inside and outside of plant cells, which could, consequently, prompt the diffusion rate of solute particles and make more saponin molecules enter the solution [13]. Statistical analysis showed that there was no significant effect of the material to solvent ratio (1:30 to 1:40 g/mL) on the total saponin content of the extracts, and more solvent used would cause a higher cost [10]. Based on the above factors, the optimized material to solvent ratio was 1:30.

#### 3.1.5. Optimization of Extraction Conditions by Box–Behnken Design

According to the optimal BBD design, the calculation equation of total saponins was as follows

Y = 124.59 + 1.56A + 0.77B − 8.51C − 2.46D − 5.55AB − 5.22AC − 0.42AD − 6.18BC + 0.62BD + 0.75CD − 7.33A^2^ − 6.98B^2^ − 14.33C^2^ − 4.80D^2^ (4)

Statistical analysis showed that the second-order polynomial model can be used to predict experimental data. From the BBD, the experimental data had an excellent correlation with the predicted data (Table 1); based on the coefficient of determination (R^2^ = 0.9209), a total of 92.09% of the variation in the total saponin content could be explained, while only 7.91% of the variation could not be explained by the model (Table S3). In addition, the lack of fit for the total saponin content was nonsignificant (*p* = 0.1969), indicating that the second-order polynomial model was adequate to describe the effects of the variables in the model. Table S3 shows that the pressure (A), pressure-holding time (B) and ethanol concentration (C) were very significant (*p* < 0.01) for the extraction of total saponins. However, there was no significant difference (*p* > 0.05) for the ratio of material to solvent (D). There were no significant interactions for A and D (*p* = 0.8460), B and D (*p* = 0.7756) or C and D (*p* = 0.7297).

Figure S1a–c exhibits the interaction effects of other factors on the output of total saponins. The optimal extractive conditions for total saponins were as follows: a pressure-holding time of 8 min, an ethanol concentration of 68%, a ratio of material to solvent of 1:35, and a pressure of 510 MPa, and the output of total saponins was 127.890 mg/g under these extractive conditions, which was almost identical to the predicted results 126.712 mg/g for total saponins. The results indicate that the model can be used to optimize the extraction process of total saponins.

### 3.2. Purification of Total Saponins under the Optimized Extraction Conditions

#### 3.2.1. Effect of [C_4_MIM]BF_4_ Concentration, Sodium Hydroxide Concentration and Material to Solvent Ratio on Purification Efficiency of Saponins by IL-Based ABS

Through single factor experiments, the purification conditions of IL-based ABS were optimized to obtain as many total saponins as possible. The effects of [C_4_MIM]BF_4_ concentration, sodium hydroxide concentration and the material to solvent ratio on the purification efficiency of saponins were investigated.

**Figure 3:**
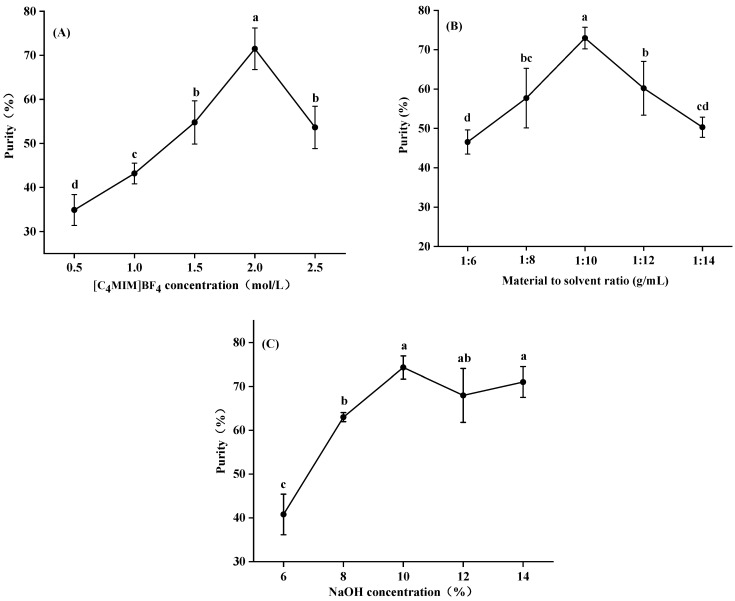
Effect of [C_4_MIM]BF_4_ concentration (**A**), material to solvent ratio (**B**) and sodium hydroxide concentration (**C**) on the purity of saponins by ionic liquid purification. Values are means of triplicate ± standard deviation. Different lowercase letters represent significant differences (*p* < 0.05).

The effects of different [C_4_MIM]BF_4_ concentrations (0.5–2.5 M) in the aqueous solution on the purification efficiency of saponins were investigated. As shown in Figure 3A, with the increase in [C_4_MIM]BF_4_ concentration from 0.5 M to 2.0 M in the purification system, the purity significantly increased. This may be due to the strong ability of [C_4_MIM]BF_4_ to destroy the cell wall and solubilize the target compounds. With the increase in concentration, the cell wall of the tissue ruptured and a large amount of saponin leached [28]. Nevertheless, when the [C_4_MIM]BF_4_ concentration was higher than 2.0 M, some of the saponins were dissolved in the upper phase of the ionic liquid, leading to a decrease in the purity of *M. charantia* saponins in the lower phase [29], which was consistent with our results.

As we know, the ratio of the material to solvent can observably influence the purification efficiency of saponins. Generally, a large volume of the [C_4_MIM]BF_4_ solution has a higher purification efficiency, and excessive solvent will cause a waste of resources. Conversely, insufficient [C_4_MIM]BF_4_ solution may lead to an incomplete extraction [12]. We can draw a conclusion from Figure 3B that with the increase in the material to solvent ratio from 1:6 to 1:14, the purity increases first and then decreases, which is consistent with the results of Tan et al. [30]. The optimal solvent to solid ratio was 1:10, which was finally adopted.

The concentration of sodium hydroxide had an important effect on the dissolution of saponins in the sodium hydroxide-rich lower phase, which may affect the purification ability. As shown in Figure 3C, the highest value (74.30%) could be achieved at 10.0% sodium hydroxide concentration. On the other side of the coin, the purity of saponins was raised markedly with the increase in the sodium hydroxide concentration from 6.0% to 10.0%. The purity had no evident variation with further increased sodium hydroxide concentration from 10.0% to 14.0%, which is in agreement with the study of Ji et al. [12], who worked out that the purity saponins could reach 81.5%. Based on the above, we determined that 10.0% sodium hydroxide was the optimal concentration.

#### 3.2.2. Optimization of Purification Conditions by BBD

Experiments on the saponins’ purity are shown in Table 2, and the calculation formula for saponin purity is as follows

Y = 74.94 + 6.41A + 4.72B − 0.51C + 0.50AB + 0.57AC − 1.32BC − 10.69A^2^ − 10.00B^2^ − 6.67C^2^ (5)

As listed in Table S4, the determined coefficient, R^2^ at 0.9819, indicates that only 1.81% of total variance was not explained by this model. The adjusted R^2^ was 0.9586, and there was a better correlation between the experimental value and the predicted value, which implies that this model was accurate and reliable for screening the purification process of saponins.

**Figure 4:**
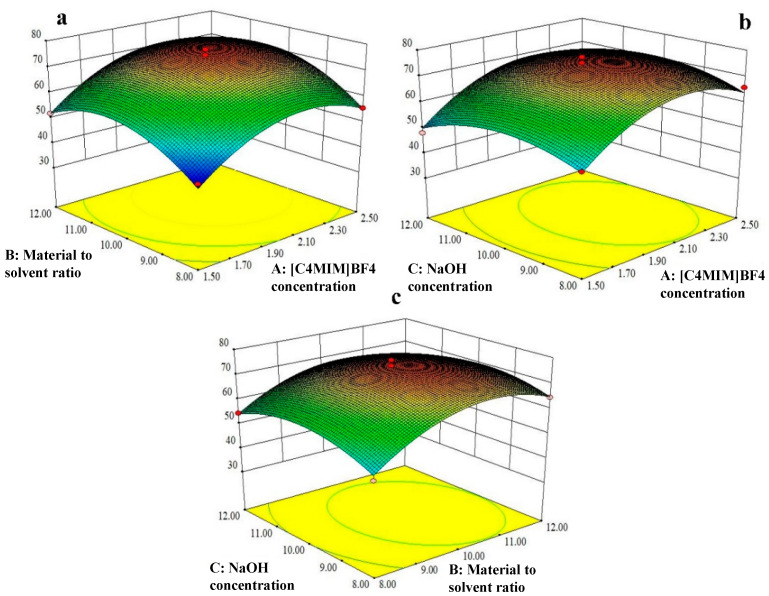
(**a**): Response surface plot of effect of [C_4_MIM]BF_4_ concentration (A, mol/L) and material to solvent ratio (B, g/mL) on the purity of saponins by ionic liquid purification; (**b**): response surface plot of effect of [C_4_MIM]BF_4_ concentration (A, mol/L) and NaOH concentration (C, %) on the purity of saponins by ionic liquid purification; (**c**): response surface plot of effect of material to solvent ratio (B, g/mL) and NaOH concentration (C, %) on the purity of saponins by ionic liquid purification.

Additionally, the linear coefficients of [C_4_MIM]BF_4_ concentration (A) and the material to solvent ratio (B) were very significant (*p* < 0.01) for the purification procedure of saponins. However, there was no significant difference (*p* > 0.05) for sodium hydroxide concentration (C). The order of influence of these three factors on the purity of saponins was A (*p* < 0.0001) > B (*p* = 0.0004) > C (*p* = 0.5206). Furthermore, the interactions of [C_4_MIM]BF_4_ concentration (A), the material to solvent ratio (B) and sodium hydroxide concentration (C) with the purity of saponins are listed in Table S4 and Figure 4; there were no significant interactions for A and B (*p* = 0.6553), A and C (*p* = 0.6099) or B and C (*p* = 0.2566).

The optimal purification conditions of saponins were as follows: a [C_4_MIM]BF_4_ concentration of 2.15 mol/L, a sodium hydroxide concentration of 9.90% and a material to solvent ratio of 1:10.5, and the purity of saponins was 76.06% under these purification conditions; the relative error with the theoretical predicted value was 0.60%. These results suggest that the model can be used for the optimization of the saponin purification processes.

### 3.3. Mass Spectral Identification of Saponins

LC/MS was utilized to detect and characterize saponins after purifying. In this study, the mass spectrometry data were analyzed based on the experimental results of LC/MS, and the Δppm value was calculated by comparing the theoretical mass charge number of the compound detected in the *M. charantia* extract with its experimental mass charge number. If the Δppm value error was between ±50 ppm, the compound was considered to be a positive match. The saponins from *M. charantia* are mainly composed of cucurbitane-type triterpenoids [31]. Due to the low UV absorption properties of cucurbitane triterpenoids, there are few commercially available standard compounds. Therefore, LC/MS is commonly used to identify the saponins from *M. charantia* [8].

More than 240 cucurbitane-type triterpenes and their glycosides have been isolated and purified from *M. charantia* in existing studies [32]. The results of mass spectrometry in this study showed that kuguaglycoside A (*m*/*z* 657.41), momordicoside L (*m*/*z* 658.45), kuguacin B (*m*/*z* 457.36) and kuguacin J (*m*/*z* 455.34) were present in the *M. charantia* extracts (Table 3). Keller et al. [6] detected five saponins from *M. charantia* extracts, including momordicine I, momordicine II and momordicoside G. The peak times of the five substances were concentrated in the range of 16.53–28.60 min, which was basically similar to the results of the present study.

## 4. Conclusions

This study investigated the extraction process, purification process and chemical compositions of saponins from *M. charantia*. The optimal extraction and purification parameters were determined by a single factor experiment and response surface methodology. IL-ABS was applied to the purification of *M. charantia* saponins, which provided new insights into their purification, and the results indicated that HHP extraction coupled with ionic liquid purification treatment is a convenient method to obtain high-purity saponins from *M. charantia*. The purification optimal experimental conditions were a [C_4_MIM]BF_4_ concentration of 2.15 M, a material to solvent ratio of 1:10.5 g/mL and a sodium hydroxide concentration of 9.90%. Moreover, the composition of saponins was preliminarily characterized by LC/MS, which indicated that kuguaglycoside A, momordicoside L, kuguacin B and kuguacin J were the main substances present in saponins. The research can help to establish a foundation for developing a simple and novel method for efficiently obtaining saponins. It can also be meaningful for bioactivity studies of saponins.

## Figures and Tables

**Table 1:** Box–Behnken experimental design and the extraction output of total saponins from *M. charantia* (*n* = 3). Each determination was made in triplicate.

No.	Pressure (MPa)	Pressure-Holding Time (min)	Ethanol Concentration (%)	Ratio of Material to Solvent (g/mL)	Output (mg/g)
1	500	6	75	1:35	99.701
2	450	8	75	1:35	97.916
3	500	8	70	1:35	123.638
4	500	8	70	1:35	128.730
5	450	8	65	1:35	108.257
6	500	8	75	1:30	102.640
7	500	8	70	1:35	123.690
8	500	8	75	1:40	101.223
9	500	8	70	1:35	120.856
10	500	6	70	1:40	103.690
11	500	10	75	1:35	86.315
12	500	10	70	1:30	118.021
13	500	10	70	1:40	111.722
14	500	6	65	1:35	107.260
15	500	10	65	1:35	118.598
16	550	8	70	1:30	117.076
17	550	10	70	1:35	109.622
18	450	8	70	1:30	111.512
19	500	6	70	1:30	112.457
20	550	8	70	1:40	111.932
21	450	8	70	1:40	108.047
22	450	10	70	1:35	113.087
23	450	6	70	1:35	103.165
24	500	8	65	1:40	110.094
25	550	6	70	1:35	121.906
26	550	8	75	1:35	84.478
27	500	8	70	1:35	126.052
28	500	8	65	1:30	114.504
29	550	8	65	1:35	115.711

**Table 2:** Box–Behnken experimental design and the purification of saponins (*n* = 3).

No.	A [C_4_MIM]BF_4_ Concentration (mol/L)	B Material to Solvent Ratio	C Sodium Hydroxide Concentration (%)	Purity (%)
1	1.5	1:8	10.0	45.28
2	1.5	1:10	8.0	52.61
3	1.5	1:12	10.0	51.95
4	2.0	1:10	10.0	77.33
5	2.0	1:10	10.0	74.98
6	2.5	1:10	12.0	63.69
7	2.0	1:10	10.0	75.15
8	2.0	1:10	10.0	74.31
9	2.5	1:12	10.0	64.21
10	2.0	1:10	10.0	72.93
11	2.0	1:12	12.0	63.20
12	1.5	1:10	12.0	48.16
13	2.5	1:8	10.0	55.55
14	2.0	1:8	12.0	54.62
15	2.0	1:8	8.0	50.71
16	2.5	1:10	8.0	65.87
17	2.0	1:12	8.0	64.56

Each determination was made in triplicate.

**Table 3:** Cucurbitane-type triterpenoids identified in *M. charantia* extracts by LC/MS.

Compound	Formula	Theoretical (*m*/*z*)	Experimental (*m*/*z*)	Adduct	Δppm
Kuguaglycoside A	C_37_H_62_O_8_	657.43	657.41	M + Na	30.42
Momordicoside L	C_36_H_58_O_9_	658.44	658.45	M + Na	−15.19
Kuguacin B	C_30_H_48_O_3_	457.34	457.36	M + H	−43.73
Kuguacin J	C_30_H_46_O_3_	455.35	455.34	M + H	21.96

## Data Availability

Data is contained within the article or Supplementary Material.

## Supplementary Materials

The following supporting information can be downloaded at: https://www.mdpi.com/article/10.3390/foods11131930/s1, Figure S1: (a): Response surface plot of pressure (A, MPa) and pressure-holding time (B, min) on the extraction output of total saponin of momordica charantia; (b): Response surface plot of pressure (A, MPa) and ethanol concentration (C, g/mL) on the extraction out-put of total saponin of momordica charantia; (c): Response surface plot of pressure-holding time (B, min) and ethanol concentration (C, g/mL) on the extraction output of total saponin of momordica charantia; Table S1: Independent variables and their levels in Box-Behnken design for total saponins of momordica charantia; Table S2: Independent variables and their code variable levels used for the Box–Behnken design; Table S3: Analysis of variance for the experimental results of the Box-Behnken design for total saponins from momordica charantia; Table S4: Analysis of variance for the experimental results of the Box-Behnken design for purification of saponins.
