# Supplementary material for: Purified Saponins in Momordica charantia Treated with High Hydrostatic Pressure and Ionic Liquid-Based Aqueous Biphasic Systems
Source: Foods. 2022 Jun 29;11(13):1930. doi: 10.3390/foods11131930 (PMC9265297; doi:10.3390/foods11131930)
Supplement: Supplementary file 1 [file foods-11-01930-s001.zip › foods-1746713-supplementary.pdf]

Table S1. Independent variables and their levels in Box-Behnken design for total saponins of momordica charantia.

Levels	A<br>Pressure（MPa）	B Pressure-holding time<br>（min）	C<br>Ethanol concentration（%）	D<br>Ratio of material to Solvent<br>(g/mL)
-1	450	6	65	1:30
0	500	8	70	1:35
1	550	10	75	1:40

Table S2 Independent variables and their code variable levels used for the Box–Behnken design.

Levels	A<br>[C4MIM]BF4 concentration（mol/L）	B<br>material to solvent ratio (g/mL)	C<br>NaOH concentration（%）
-1	1.5	1:8	8.0
0	2.0	1:10	10.0
1	2.5	1:12	12.0

Table S3 Analysis of variance for the experimental results of the Box-Behnken design for total saponins from momordica charantia.

Variables	Sum of squares	DF	Mean square	F value	P value Prob ＞F
Model	2935.256	14	209.661	11.640	< 0.0001
A<br>Pressure	478.190	1	478.190	26.548	0.0001
B<br>Pressure-holding time	392.448	1	392.448	21.787	0.0004
C<br>Ethanol concentration	1330.159	1	1330.159	73.846	< 0.0001
D<br>Ratio of material to solvent	29.016	1	29.016	1.611	0.2251
AB	123.262	1	123.262	6.843	0.0203
AC	109.123	1	109.123	6.058	0.0274
AD	0.705	1	0.705	0.039	0.8460
BC	152.824	1	152.824	8.484	0.0114
BD	1.522	1	1.522	0.084	0.7756
CD	2.238	1	2.238	0.124	0.7297
A^2	348.538	1	348.538	19.350	0.0006
B^2	315.657	1	315.657	17.524	0.0009
C^2	1332.287	1	1332.287	73.964	< 0.0001
D^2	149.699	1	149.699	8.311	0.0120
Residual	252.176	14	18.013		
Lack of Fit	217.239	10	21.724	2.487	0.1969
Pure Error	34.937	4	8.734		
Cor total	3187.4319	28			
R^2	0.9209			0.9209	
Adj R^2	0.8418			0.8418	

Adj: adjusd.

Table S4 Analysis of variance for the experimental results of the Box-Behnken design for purification of saponins.

Variables	Sum of squares	DF	Mean square	F value	P value Prob ＞F
Model	1730.14	9	192.24	42.12	< 0.0001
A [$C_4MIM]BF_4$ concentration	329.12	1	329.12	72.11	< 0.0001
B Material to solvent ratio	178.21	1	178.21	39.05	0.0004
C NaOH concentration	2.09	1	2.09	0.46	0.5206
AB	0.99	1	0.99	0.22	0.6553
AC	1.30	1	1.30	0.29	0.6099
BC	6.96	1	6.96	1.53	0.2566
A^2	481.40	1	481.40	105.48	< 0.0001
B^2	421.15	1	421.15	92.28	< 0.0001
C^2	187.25	1	187.25	41.03	0.0004
Residual	31.95	7	4.56		
Lack of Fit	21.74	3	7.25	2.84	0.1697
Pure Error	10.21	4	2.55		
Cor total	1762.09	16			
R^2	0.9819				
Adj R^2	0.9586				

Adj: adjusd.

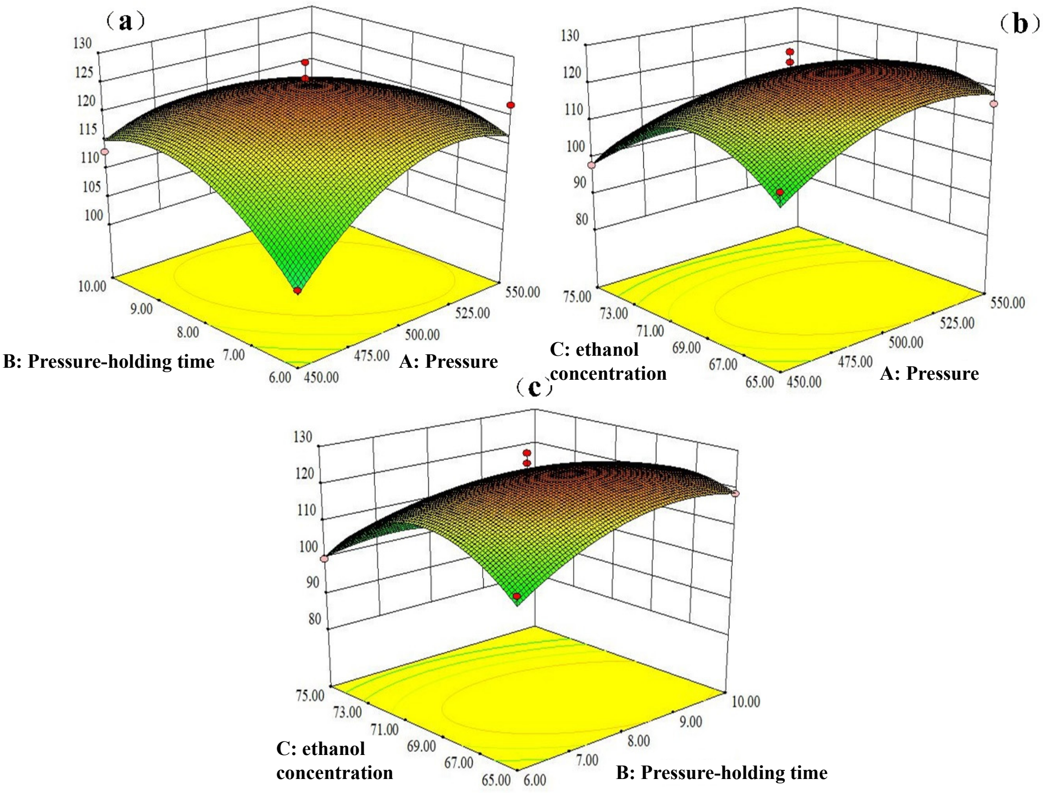

Figure S1. (a): Response surface plot of pressure (A, MPa) and pressure-holding time (B, min) on the extraction output of total saponin of momordica charantia; (b): Response surface plot of pressure (A, MPa) and ethanol concentration (C, g/mL) on the extraction output of total saponin of momordica charantia; (c): Response surface plot of pressure-holding time (B, min) and ethanol concentration (C, g/mL) on the extraction output of total saponin of momordica charantia.
